# Structural and Physicochemical Characterization of Rhamnolipids produced by *Pseudomonas aeruginosa* P6

**DOI:** 10.1186/s13568-020-01141-0

**Published:** 2020-11-04

**Authors:** Ghadir S. El-Housseiny, Khaled M. Aboshanab, Mohammad M. Aboulwafa, Nadia A. Hassouna

**Affiliations:** 1 Department of Microbiology and Immunology, Faculty of Pharmacy, Organization of African Unity St, Ain shams University, POB: 11566, Abbassia, Cairo, Egypt; 2 Faculty of Pharmacy, King Salman International University, Ras-Sedr, South Sinai Egypt

**Keywords:** Biosurfactants, Stability, Critical micelle concentration, Bioremediation, Glycerol, Di-rhamnolipids

## Abstract

Rhamnolipids are important biosurfactants for application in bioremediation, enhanced oil recovery, pharmaceutical, and detergent industry. In this study, rhamnolipids extracted from *P. aeruginosa* P6 were characterized to determine their potential fields of application. Thin-layer chromatographic analysis of the produced rhamnolipids indicated the production of two homologues: mono- and di-rhamnolipids, whose structures were verified by ^1^H and ^13^C nuclear magnetic resonance spectroscopy. Additionally, high performance liquid chromatography-mass spectrometry identified seven different rhamnolipid congeners, of which a significantly high proportion was di-rhamnolipids reaching 80.16%. Rha-Rha-C10-C10 was confirmed as the principal compound of the rhamnolipid mixture (24.30%). The rhamnolipids were capable of lowering surface tension of water to 36 mN/m at a critical micelle concentration of 0.2 g/L, and exhibited a great emulsifying activity (E24 = 63%). In addition, they showed excellent stability at pH ranges 4–8, NaCl concentrations up to 9% (w/v) and temperatures ranging from 20 to 100 °C and even after autoclaving. These results suggest that rhamnolipids, produced by *P. aeruginosa* P6 using the cheap substrate glycerol, are propitious for biotechnology use in extreme and complex environments, like oil reservoirs and hydrocarbon contaminated soil. Moreover, *P. aeruginosa* P6 may be considered, in its wild type form, as a promising industrial producer of di-RLs, which have superior characteristics for potential applications and offer outstanding commercial benefits.

## Introduction

Rhamnolipids (RLs) are a large family of well-studied glycolipid biosurfactants predominantly produced by *Pseudomonas aeruginosa* (*P. aeruginosa*) and other *Pseudomonas* spp. (Lotfabad et al. 2010). They are regarded as one of the most promising classes of biosurfactants as a result of their limited toxicity, powerful surface activity and emulsifying activity, biodegradability and environmentally friendly nature (Shatila et al. 2020). Accordingly, they may be used in various industries like pharmaceuticals, cosmetics, food and agriculture. Moreover, they are also used in enhanced oil recovery (EOR) and bioremediation of heavy metals and hydrocarbon pollutants (Zhao et al. 2019). The use of chemical surfactants for enhancing oil recovery and biodegradation is unfavorable as they are toxic, expensive and may negatively impact the environment. The use of microbial biosurfactants has proven to be a preferable option, however, this relies on their performance at drastic conditions of temperature, salinity and pH (Gogoi et al. 2016). Previous reports studied the effect of exogenously seeding biosurfactant producing microbes on bioremediation of crude oil contaminated fields (Das and Mukherjee 2007).

RLs consist of 1 or 2 β-hydroxyl (3-hydroxy) fatty acids attached by an *O*-glycosidic linkage to 1 or 2 rhamnose sugar molecules. Based on the number of rhamnose units, they are classified into mono-rhamnolipids (mono-RLs) and di-rhamnolipids (di-RLs) (Zhou et al. 2019). The hydroxyacyl part varies with typical chain lengths ranging from eight to sixteen (mainly a chain length of ten carbons in *P. aeruginosa)* (Behrens et al. 2016; Gunther et al. 2005). The structural composition of RLs is highly diverse and is greatly affected by the producing strains and cultivation conditions (Rahman et al. 2010). Several studies showed that RLs can consist of a combination of 4–28 different congeners (Lotfabad et al. 2010; Monteiro et al. 2007; Zhou et al. 2019). Differences in RL composition affect their surface and emulsifying activities. The different physicochemical properties of RLs will consequently decide their potential application in diverse fields (Zhao et al. 2018).

*Pseudomonas aeruginosa* P6 is a promising RL producing strain isolated, identified and optimized for maximum RL production using glycerol containing media (GMSM) in our previous studies (El-Housseiny et al. 2016, 2017). Therefore, the goal of this study was to structurally characterize the RLs produced by this isolate, using thin layer chromatography (TLC), nuclear magnetic spectroscopy (NMR) and high performance liquid chromatography-mass spectrometry (HPLC-MS). Physicochemical characterization was also carried out by determining its ability to reduce surface tension (ST), its critical micellar concentration (CMC), its emulsifying activity and also its stability in regards to different temperatures, NaCl concentrations and pH values. The results of RL homolog composition and their characteristics will determine the potential use of the RL products and the need for optimization of the relative product components.

## Materials and methods

### Microorganism

*P. aeruginosa* P6 from Culture Collection Ain Shams University (CCASU) (strain number, CCASUP6) is a promising RL producer obtained in our previous study through a screening program (El-Housseiny et al. 2017). It was preserved in Luria–Bertani broth (LB broth) with 20% glycerol at − 80 °C.

### Culture media

The GMSM (mineral salts medium (Bodour et al. 2003) with 2% v/v glycerol added as the only carbon source, pH 7.5) was chosen as the optimum media for maximum production of RLs in our previous study (El-Housseiny et al. 2016). Therefore, it was prepared and used in this study.

### Production of RLs

A loopful from a fresh culture of *P. aeruginosa* P6 on trypticase soy agar slant was inoculated into 25 mL trypticase soy broth (TSB) and incubated at 30 °C and 250 rpm overnight to obtain seed culture. The bacterial cells obtained after centrifugation for 10 min at 10,000 rpm (corresponding to a relative centrifugal force of 7380×*g*) were then washed and resuspended in GMSM to get a count of 5 × 10^9^ cfu/mL.

The production process was carried out using optimum conditions described in our previous study (El-Housseiny et al. 2016). Briefly, 5% v/v of the seed culture was used to inoculate 50-mL aliquots of GMSM (containing 2% v/v glycerol as the carbon source) and incubated at 250 ± 2 rpm and 30 °C for 6 days.

### Extraction of RLs

Culture broth was centrifuged (7380×*g* for 10 min) to obtain the cell free supernatant (CFS). This CFS was acidified to pH 2 for 18 h at 4 °C. Then, equal volumes of ethyl acetate were vigorously shaken twice with the acidified CFS to extract RLs. Finally, the ethyl acetate layers were collected and evaporated at 80 °C using a rotary evaporator to obtain a honey-colored residue (Wu and Ju 1998). This residue was used as such or dissolved in 10 mL distilled water set to pH 7 using 2.5 N NaHCO_3_ (called aqueous RL solution) for further studies.

### Structural characterization of RLs

The extracted RLs were investigated for their chemical structure by TLC, NMR spectroscopy and HPLC-MS analysis. All spectroscopic measurements were performed at Drug Discovery and Development Research Center at Faculty of Pharmacy, Ain Shams University, Abbassia, Cairo, Egypt.

### TLC analysis

TLC experiments were performed as explained by Lotfabad et al. (2010). The residue obtained after RL extraction was dissolved in methanol and 10 µl was loaded on a TLC plate (5 × 10 cm) (silica gel 60, Sigma, USA). After drying, chloroform: methanol: water (6.5: 2.5: 0.4 v/v/v) was used as the solvent system to develop the plate. After that, the plate was left to air-dry then it was evenly sprayed with orcinol reagent (0.19% orcinol in 53% H_2_SO_4_). Finally, the plate was placed in an oven at 120 °C for 15 min and the migration distances of the obtained colored spots were measured and their corresponding R_f_ values were calculated.

### NMR spectrometry

^1^H NMR and ^13^C NMR spectra of the extracted RLs were measured using a Bruker® Avance III HD (400 MHz) spectrometer (Germany) equipped with a 5 mm broad-band multinuclear (PABBO) probe with deuterated methanol (CD30D) as a solvent.

### HPLC–MS analysis

The residue of the extracted RLs was dissolved and diluted in methanol to be analyzed by HPLC-MS. The analysis was performed using LCMS-8045 Triple Quadrupole Liquid Chromatograph Mass Spectrometer (LC–MS/MS, Nexera series, Shimadzu) equipped with a C18 reversed phase column (Waters UPLC, particle size of 1.7 microns). The detector was a LC-2030/2040 PDA Detector with an electrospray ionization interface (ESI). All RLs were detected in the negative ion mode producing [M–H]- pseudomolecular ions.

The analysis was carried out with a sample injection volume of 20 µL, mobile phase at a flow rate of 0.2 mL/min with a gradient of water (A): acetonitrile (B) (with 0.1% formic acid) from 10 to 95% in 28 min. The gradient was adjusted to 10% B from 0 to 2 min, increased to 90% B from 2 to 20 min and remained constant for 5 min at 90% B before it decreased to 10% in 2 min and remained constant for equilibration. The mass spectrometer was in the negative ESI mode and the scanning mass range was from 200 to 900 m/z (mass to charge ratio). RL congeners were identified based on m/z and their relative proportions were calculated using the obtained area for each congener of RL by HPLC–MS (Haba et al. 2014; Zhao et al. 2019).

### Physicochemical characterization of RLs

### Determination of ST and CMC

Determination of ST (mN/m) of the CFS and aqueous RL solution was carried out using a Stalagmometer by Drop count method (Joe et al. 2019; Kaya et al. 2014). The ST was calculated using the following formula:

$$\begin{gathered} \sigma _{{\text{L}}} = {\text{ }}\sigma _{{\text{W}}} \times \underline{{{\text{Nw}}}} \times \underline{{\rho _{{\text{L}}} }} \hfill \\ \quad \quad \quad \quad \;\;{\text{N}}_{{\text{L}}} \quad \rho _{{\text{W}}} \hfill \\ \end{gathered}$$

where, σ_L_ and N_L_ is the ST and the number of drops of the aqueous RL solution, respectively. σ_W_ and N_W_ is the ST and the number of drops of water, respectively. ρ_L_ is the density of the RL solution, and ρ_W_ is the density of water.

To determine CMC, the aqueous RL solution was serially diluted (1000–0 mg/L), and the ST of these dilutions were obtained. Experiments were performed in triplicates at room temperature and mean values recorded. The CMC of the RL solution was obtained from the breakpoint of the ST plotted versus RL concentration (Câmara et al. 2019; Kaya et al. 2014; Khademolhosseini et al. 2019).

### Emulsifying activity

The emulsifying activity of CFS was obtained by calculating the emulsification index (E24) using hexadecane (Abdel-Mawgoud et al. 2009). Two mL of hexadecane were mixed with 2 mL of the CFS followed by vortexing for 2 min. After 24 h, E24 was calculated as shown below (Iqbal et al. 1995):

E24 = (height of emulsion layer/height of total mixture) x 100.

### Oil spreading test

RL activity was determined by Oil Spreading Technique (OST) (Morikawa et al. 1993). Briefly, 20 µl crude oil was placed onto the surface of 40 mL distilled water in a 140 mm petri dish to develop a thin membrane of oil. Five µl of the CFS were then carefully dropped onto the center of the oil membrane. The diameters of the clear halos produced were measured. All tests were performed in triplicates at room temperature and mean values recorded.

### Evaluation of the stability of RLs

The surface activity of 0.3 g/L RL solution was determined using the OST at room temperature (25 °C) as described above. This served as the control. After that, a set of RL solutions were subjected to the different conditions explained below. The dimensionless diameter was calculated by dividing the diameters obtained by the maximum diameter obtained from the control (Khademolhosseini et al. 2019). Statistical analysis was carried out in GraphPad Prism V.8.4.3 using a one-way ANOVA; the significance of the results was tested at p < 0.05 level.

### Different temperatures

To assess the thermal stability, a set of RL solutions (0.3 g/L) were incubated at temperatures of 4, 60, 80 and 100 °C for 30 min and at 121 °C in an autoclave for 15 min, then cooled and OST was carried out (Khademolhosseini et al. 2019; Zhao et al. 2018). In addition, the effect of autoclaving on the emulsifying activity of RLs was also evaluated by calculating the E24 of CFS before and after autoclaving.

### Different NaCl concentrations

A set of RL solutions (0.3 g/L) were prepared containing different concentrations of NaCl (0, 1, 3, 6, 9 and 12% w/v), and the surface activity of these solutions was determined using OST (Khademolhosseini et al. 2019).

### Different pH

A set of RL solutions (0.3 g/L) were prepared to have pH values from 2 to 12 using 1 N HCl or 1 N NaOH and OSTs were carried out (Kaya et al. 2014; Khademolhosseini et al. 2019).

## Results

### Structural characterization of RLs

#### TLC analysis

After development of the plate and spraying with orcinol reagent, it showed two spots, one major spot and another minor one with R_f_ values of 0.56 and 0.71, respectively. These results suggest that *P. aeruginosa* P6 produced both mono-RLs and di-RLs which are the two main types of RLs produced by *P. aeruginosa* species (Lang and Wullbrandt 1999; Maier and Soberon-Chavez 2000). The higher spot is from the mono-RLs, whereas the lower spot comes from the di-RLs (Lotfabad et al. 2010).

#### NMR spectroscopy

**Fig. 1:**
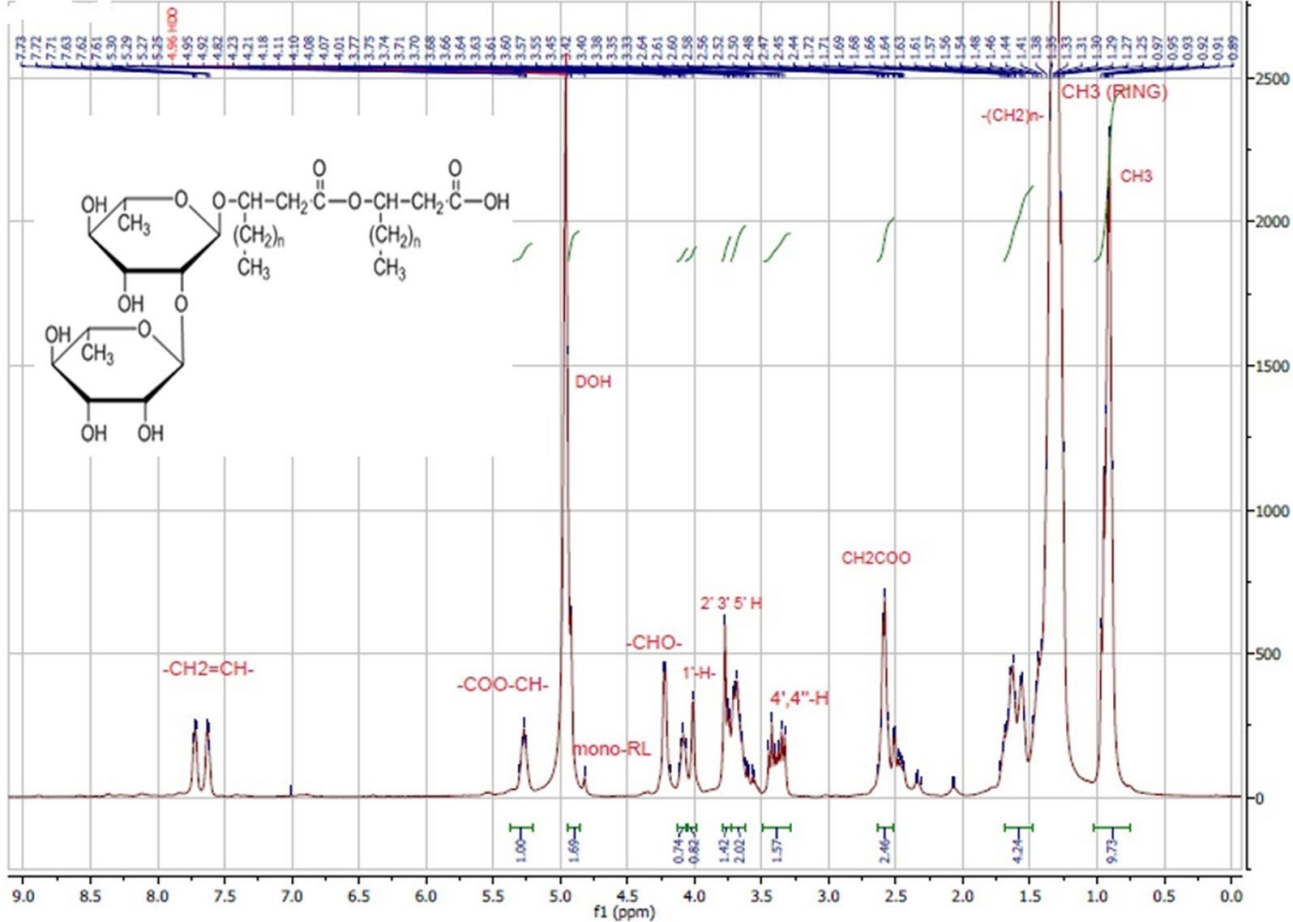
^1^H NMR spectrum of the RLs extracted from *P. aeruginosa* P6. At 4.8 ppm the small peak labelled mono-RL is due to the rhamnose anomeric proton of mono-RL species (Nicolo et al. 2017). The structure of the proposed RLs is shown over the spectrum

The ^1^H NMR spectrum of the RLs extracted from *P. aeruginosa* P6 grown in GMSM is shown in Fig. 1. Data from previous studies was used to assign the NMR signals as shown in Table 1 (Nicolo et al. 2017; Sharma et al. 2015).

The characteristic chemical shifts revealed that the extract had the molecular structure of RL homologues. The structure of the proposed RL is shown over the spectrum (Fig. 1).

**Table 1:** Chemical shift assignments of extracted RLs in ^1^H NMR spectrum produced by *P. aeruginosa* P6

Assignment	Chemical shift (ppm)
–CH_3_	0.89
–(CH_2_)n–	1.26
–CH_2_–COO–	2.4–2.6
–O–CH–	4.2
–COO–CH–	5.3
CH_3_ (ring)	1.23
4’,4’’-H	3.4
2’-,3’-,5’-H 2’’,3’’,5’’ H	3.6–3.7
1’,1’’ H	4.1
-CH_2_ = CH-	7.6–7.7

**Fig. 2:**
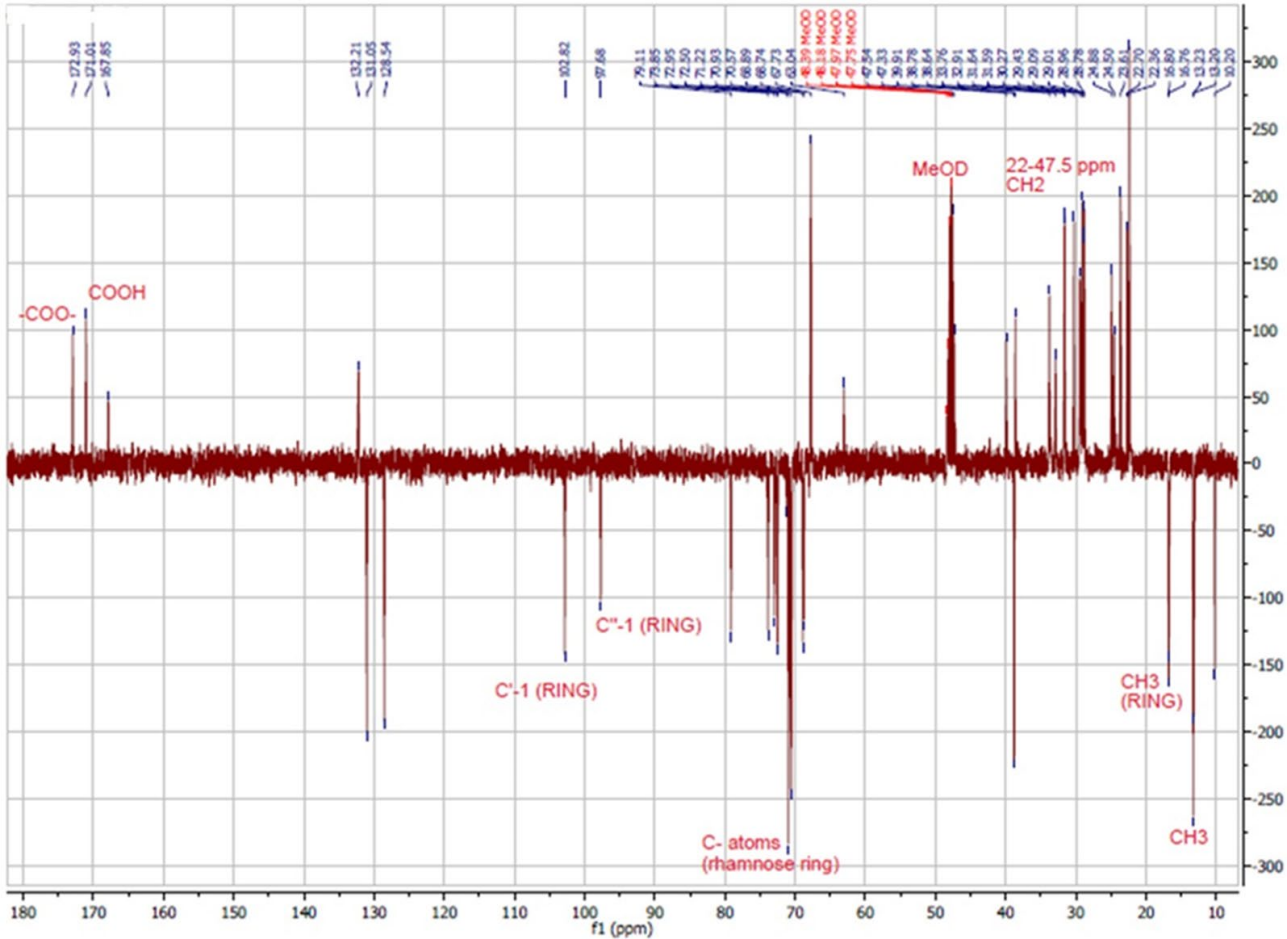
^13^C NMR spectrum (APT) of the RLs extracted from *P. aeruginosa* P6

Figure 2 shows the 13C NMR Attached Proton Transfer (APT) spectrum of the extracted RLs. The spectrum presents the signals as a function of the phase or the number of hydrogen nuclei bonded to carbon (Silva et al. 2014). CH3 and CH nuclei have a negative phase, whereas CH2 and C nuclei have a positive phase. As shown in the figure, signals with a negative phase at 13.2 and 16.9 ppm were ascribed to the 2 methyl groups (CH3) in the aliphatic chain and 2 methyl groups in rhamnose rings, respectively. The signals with a positive phase between 22.0 and 47.5 ppm were attributed to methylene groups (CH2); signals with a negative phase at 97.68 and 102.82 ppm were due to two C-1 carbons of two rhamnose units. Signals with a positive phase at 171 and 172.93 ppm were attributed to the carboxylic acid and ester, respectively. All signals with a negative phase in the range of 68.74–79.11 ppm were attributed to carbon atoms in the rhamnose moiety. NMR spectral analysis of the mixture were assigned to a typical RL-type structure (Charles Oluwaseun et al. 2017; Christova et al. 2013; Silva et al. 2014; Twigg et al. 2018).

#### HPLC–MS analysis

**Fig. 3:**
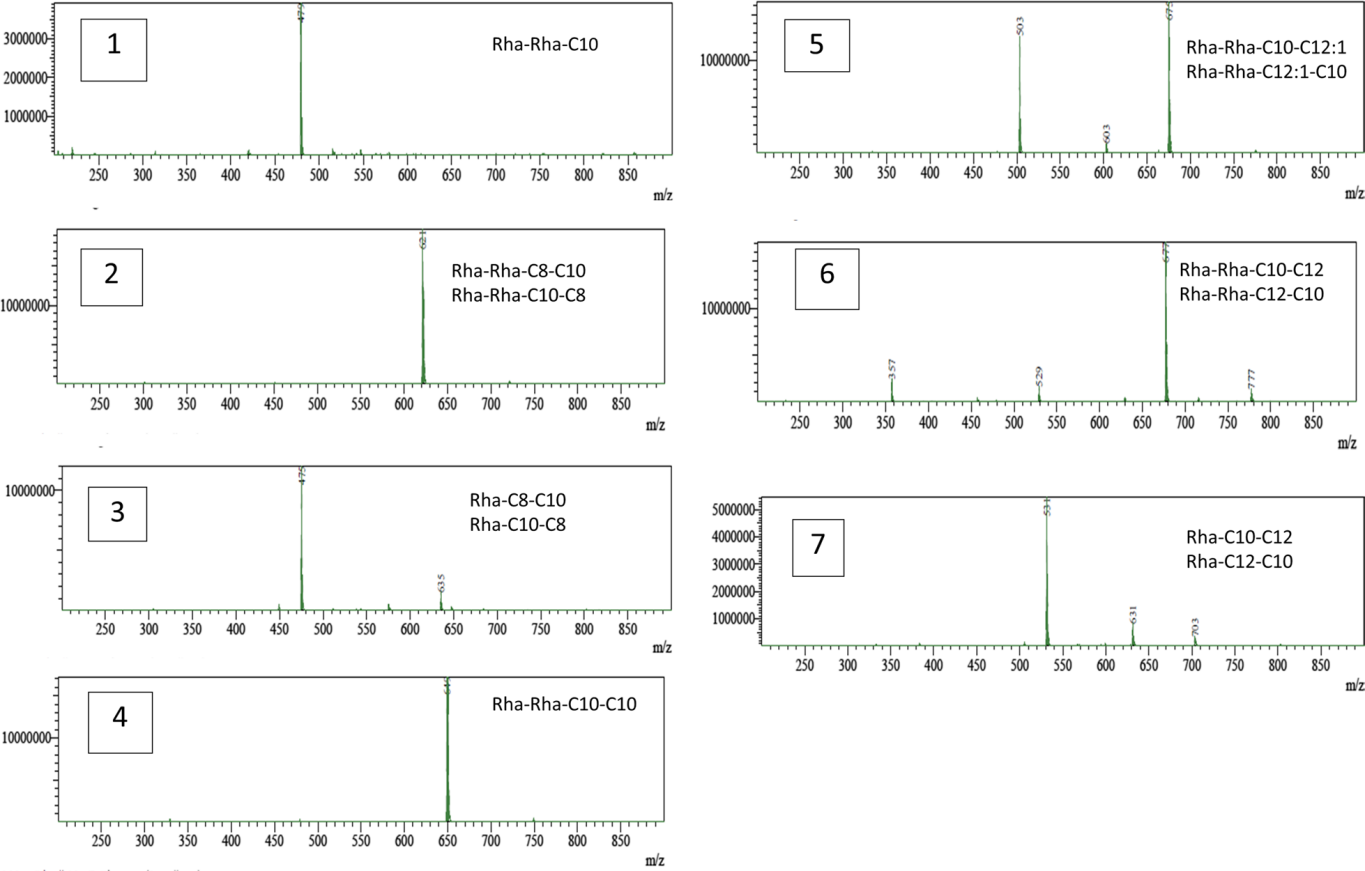
MS spectra of the RLs extracted from *P. aeruginosa* P6. Top right corner shows the suspected formula of the RLs. X- and Y-axis indicate m/z (mass to charge ratio) and intensity, respectively

The HPLC–MS profile obtained from the extract showed the presence of predominant peaks corresponding to the molecular weight of 7 RL homologues: 2 mono-RLs and 5 di-RLs (Fig. 3). The 2 mono-RL homologues may exist as 2 isoforms (Rha–C_8_–C_10_/Rha–C_10_–C_8_ and Rha–C_10_–C_12_/Rha–C_12_–C_10_). On the other hand, 3 of the di-RL homologues may exist as isoforms (Rha–Rha–C_8_–C_10_/ Rha–Rha–C_10_–C_8_, Rha–Rha–C_10_–C_12:1_/Rha–Rha–C_12:1_–C_10_, Rha–Rha–C_10_–C_12_/Rha–Rha–C_12_–C_10_) while 1 homologue had equivalent alkyl chain length (Rha–Rha–C_10_–C_10_), and 1 homologue had only 1 alkyl chain (Rha–Rha–C_10_).

The relative abundance of each congener was calculated from the areas obtained for each molecule of RL by HPLC–MS (Gogoi et al. 2016; Haba et al. 2014). As shown in Table 2, the ratio of di-RLs to mono-RLs was 80.16: 19.84, and Rha-Rha-C_10_-C_10_ (2-*O*-α-1,2-L-rhamnopyranosyl-α-L-rhamnopyranosyl-β-hydroxydecanoyl-β-hydroxydecanoate) was the major molecule in the mixture, accounting for 24.3% of the total RLs.

**Table 2:** Composition of RL Mixture produced by *P. aeruginosa* P6

No.	[M-H]− (m/z)	RL components	Molecular formula	Relative abundance (%)	Rank (according to relative abundance)
1	479.30	Rha–Rha–C_10_	C_22_H_40_O_11_	3.06	7
2	621.40	Rha–Rha–C_10_–C_8_ Rha–Rha–C_8_–C_10_	C_24_H_44_O_9_	16.62	4
3	475.35	Rha–C_10_–C_8_ Rha–C_8_–C_10_	C_24_H_44_O_9_	9.11	6
4	649.45	Rha–Rha–C_10_–C_10_	C_32_H_58_O_13_	24.30	1
5	675.50	Rha–Rha–C_12:1_–_C10_ Rha–Rha–C_10_–C_12:1_	C_34_H_60_O_13_	18.81	2
6	677.50	Rha–Rha–C_12_–C_10_ Rha–Rha–C_10_–C_12_	C_34_H_62_O_13_	17.37	3
7	531.45	Rha–C_10_–C_12_ Rha–C_12_–C_10_	C_28_H_52_O_9_	10.73	5

#### Determination of ST and CMC

**Fig. 4:**
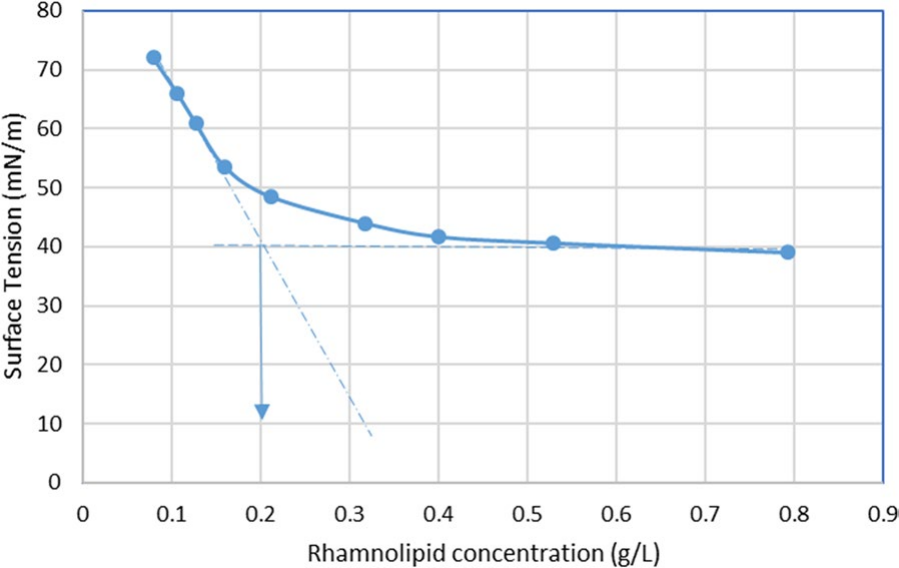
A plot of surface tension as a function of concentration of aqueous RL solution

Both the CFS and aqueous RL solution were capable of lowering water ST from 72 mN/m to 36 mN/m. The CMC was estimated from a graph plotted between RL concentration and ST obtained from the intersection of the straight line passing through the plateau with the regression straight line of the linearly dependent section. As depicted in Fig. 4, the CMC was estimated to be 200 mg/L.

#### Emulsifying activity

Emulsifying activity was determined by calculating the emulsification index (E24) using hexadecane. The E24 of the RL containing CFS with hexadecane was found to be 63.3% ± 0.06.

#### OST

RL activity was determined using the OST. Five microliters of the CFS resulted in a clear halo with a diameter of 9.17 ± 0.29 mm.

### Stability of the extracted RLs

#### Different temperatures

**Fig. 5:**
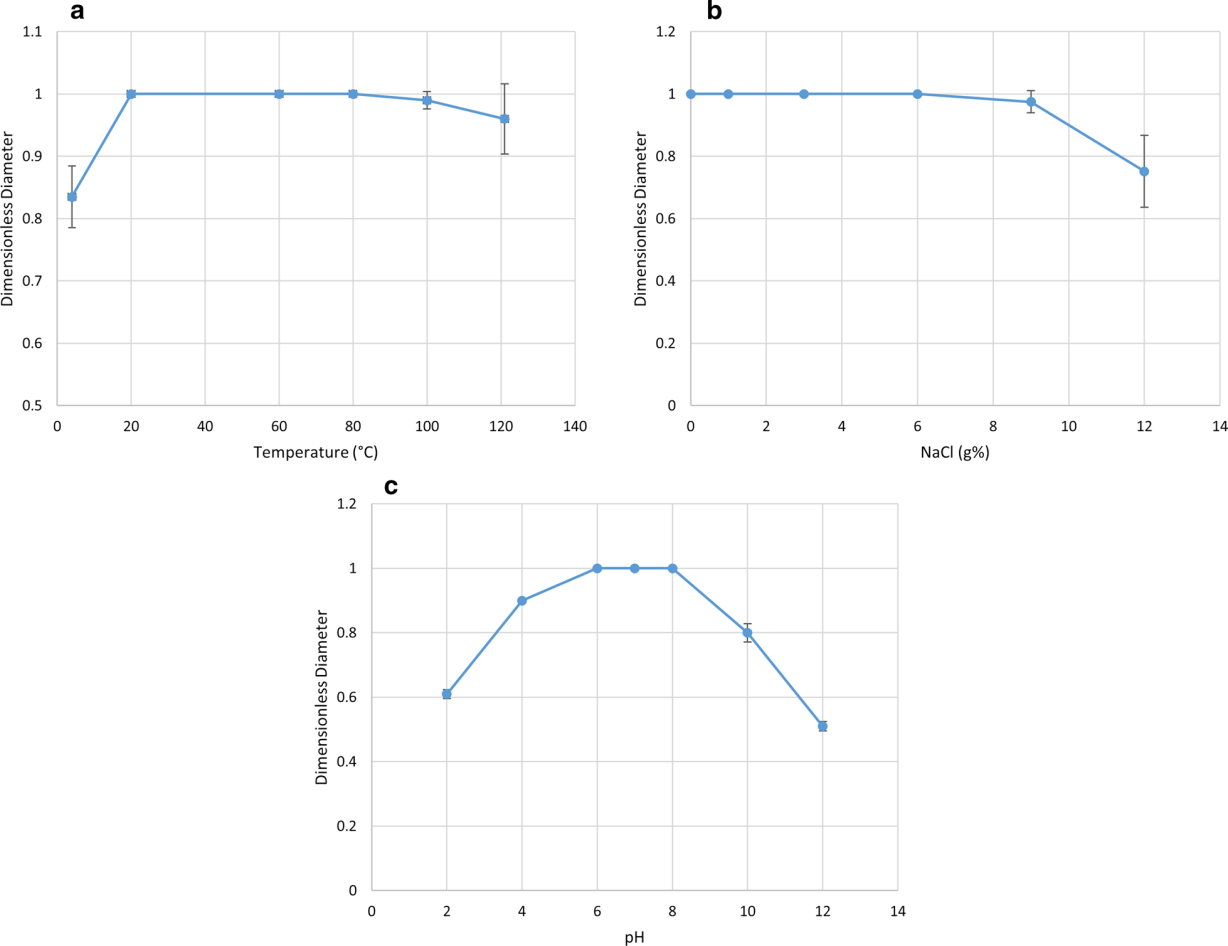
Effect of **a** temperature, **b** salinity, **c** pH on the stability of the produced RLs

As shown in Fig. 5A, the dimensionless diameter was reduced at 4 °C. However, exposure to temperatures up to 100 °C for 30 min did not alter the clear zone diameter. Moreover, the dimensionless diameter was nearly constant upon autoclaving at 121 °C for 15 min. Results were found to be statistically significant (P = 0.01).

In addition, the E24 of the RL containing CFS was slightly reduced from 63.3 to 56.7% ± 0.05 after autoclaving.

#### Different NaCl concentrations

As observed in Fig. 5b, the diameter of the clear zone was not altered by NaCl concentrations up to 9% (w/v). However, it was reduced as the salinity was increased to 12% (w/v) (P = 0.019).

#### Different pH

As depicted in Fig. 5c, the dimensionless diameter was almost constant at pH 4–8. However, it was reduced to 60% at pH 2, and further to 50% at a higher pH value of 12 (P < 0.0001).

## Discussion

RLs have attracted increasing interest in many industrial sectors, particularly in bioremediation and oil industry, which are aimed at environmentally friendly and sustainable policies. RLs’ physicochemical characters are decided by their structural composition, which in turn determines their application potential (Gogoi et al. 2016). Therefore, the aim of this study was to structurally and physicochemically characterize RLs produced by the promising isolate *P. aeruginosa* P6 using the low-cost substrate, glycerol. RLs were extracted from culture broths when maximum RL production was reached, i.e. after 6 days of incubation resulting in a maximum production of 7.54 g/L as determined previously (El-Housseiny et al. 2016).

### Structural characterization

#### TLC analysis

RLs are a series of glycolipid structures that are composed of 1 or 2 L-rhamnose sugars and 1 or 2 β-hydroxyalkanoic acids. Previous studies reported that different strains produced RLs with different compositions (Abdel-Mawgoud et al. 2010). Primary characterization was carried out using TLC. The TLC indicated the presence of two main compounds i.e. upper and lower one. The lower major spot consisted of di-RLs with R_f_ of 0.56 while the higher minor spot consisted of mono-RLs with R_f_ of 0.71. These findings are consistent with other reported studies, where Abdel-Mawgood et al. obtained a lower spot with an R_f_ value of 0.4 and a higher spot with an R_f_ value of 0.68 corresponding to di- and mono-RLs, respectively (Abdel-Mawgoud et al. 2007). Another report obtained a lower spot consisting of di-RLs with R_f_ of 0.59 and a higher spot consisting of mono-RLs with R_f_ of 0.62 for strain C1501 (Charles Oluwaseun et al. 2017). Moreover, in another study, one major lower spot from the di-RL structure showed an R_f_ value of 0.31, while the higher spot for mono-RLs had an R_f_ value of 0.76 (Lotfabad et al. 2010).

#### NMR spectroscopy

The chemical structure of the RLs synthesized by *P. aeruginosa* P6 was elucidated by analyzing the extract using NMR spectroscopy. NMR spectroscopy is an important technique for structural elucidation. In ^1^H NMR, the characteristic chemical shifts in the 0.8–1.4 and 3.3–5.5 ppm regions indicated the existence of long hydrocarbon chains and rhamnose rings, respectively (Khademolhosseini et al. 2019). In ^13^C NMR, signals between 10.0 and 40.0 ppm were ascribed to aliphatic groups; between 68 and 80 ppm were due to carbon atoms in rhamnose moiety; and signals at 170–175 ppm were attributed to the carbonyl groups (Silva et al. 2014). Taken together, ^1^H and ^13^C NMR spectra obtained proved the presence of RLs in the mixture and the spectra coincided with previously published RLs spectra for both ^1^H NMR (Charles Oluwaseun et al. 2017; Raza et al. 2009; Sharma et al. 2015) and ^13^C NMR (Charles Oluwaseun et al. 2017; Silva et al. 2014; Twigg et al. 2018). Since the precise RLs structure was unachievable using NMR, further characterization using HPLC-MS was carried out.

#### HPLC–MS analysis

Characterizing a certain RL homologue by its retention time together with its mass spectral signature can be provided by direct coupling of reverse phase liquid chromatography to a mass spectrometer. Before mass analysis, RLs are ionized mainly by Electrospray Ionization (ESI) (Abdel-Mawgoud et al. 2011). MS with ESI is therefore a powerful analytical approach which has been frequently used for the analysis of RLs, since it permits the analysis of small sized samples, even if existing in mixtures and without derivatization (Pereira et al. 2012).

In this study, the molecular composition of the extracted RLs was analyzed using ESI-MS. RLs’ identities were confirmed by detection of the masses of the individual RL congeners. Figure 3 shows the mass spectra of ion scans for each RL congener. The results showed the production of 7 RL congeners by *P. aeruginosa* P6 during growth in GMSM. Table 2 lists the different RL congeners produced in order of their elution. As shown in the results, compounds with longer fatty acid chains eluted after shorter fatty acid chain compounds as a consequence of their higher hydrophobicity. Saturated fatty acids are also more hydrophobic and therefore show more retention than their unsaturated counterparts. The congeners were ranked according to their relative abundance, calculated from their peak areas, but it was not possible to differentiate between constitutional isomers, like Rha-C10-C8 vs. Rha-C8-C10, based on chromatography (Behrens et al. 2016). However, it has been suggested that the isomer containing a shorter chain closer to the rhamnose moiety will be the dominant isomer (Deziel et al. 2000).

As delineated in Table 2, the RL homologues included both mono- and di-RLs, but di-RLs were produced in a significantly higher proportion (di-RLs: mono-RLs = 80.16:19.84) and the di-RL Rha-Rha-C10-C10 at m/z 649 was identified as the major component. This work is in agreement with previous reports in which di-RLs were the major species (Lotfabad et al. 2010; Moussa et al. 2014) with Rha-Rha-C10-C10 predominating (Rudden et al. 2015), while a few studies (Arino et al. 1996; Sim et al. 1997) reported mono-RLs as the predominant component. The results also indicate that *P. aeruginosa* P6 primarily produced di-RLs when grown in the glycerol containing media, GMSM. Similar findings were reported by other authors, who showed that when hydrophilic carbon sources like glycerol were utilized, the di-RL Rha-Rha-C10-C10 predominated (Li et al. 2019; Nicolo et al. 2017; Nitschke et al. 2005a). The results also indicated that *P. aeruginosa* P6 had an outstanding di-RLs production capability in comparison to earlier studies, since all di-RL homologues summed up to 80.16% of the total homologues in the RL mixture. Many reports have shown that most *P. aeruginosa* strains produce di-RLs in the range between 23 and 77% of the total RLs mixture (Arino et al. 1996; Lotfabad et al. 2010). These results suggest that *P. aeruginosa* P6 is a high di-RL-producing strain. The *rhlC* genes (which are responsible for di-RL production in *P. aeruginosa*) together with the carbon source, cultivation conditions, and analytical method used, may influence the ratio of mono- and di-RLs (Behrens et al. 2016; Nicolo et al. 2017). Therefore, the mechanism of high di-RL production in *P. aeruginosa* P6 deserves further study.

Di-RLs were reported to have better features than mono-RLs in several previous reports. Zhang et al. (1997) proved that phenanthrene was more bioavailable in di-RL solutions than in mono-RL. Hence di-RLs appear to be more suitable for remediation of hydrocarbon contaminated environments. In addition, di-RLs are also known to be valuable in dermatology and other clinical applications (Lotfabad et al. 2010; Rikalovic et al. 2013). Di-RLs were also reported to be more useful than mono-RLs for triclosan desorption from sediments (Zhang et al. 2013), which proved that di-RLs had a more powerful surface activity than mono-RLs. Additionally, Rikalovic et al. (2013) stated that mono-RLs begin micelle formation at concentrations lower than di-RLs, but di-RLs have higher bioavailability than mono-RLs. Finally, Li et al. (2019) proved that di-RLs would be a better choice in Microbial enhanced oil recovery (MEOR) application than mono-RLs. Considering these desirable features of di-RLs, their enhanced production is favorable.

#### Determination of ST, CMC and emulsifying activity

Our results proved the high efficiency of the produced RLs since they could reduce the water ST from 72 to 36 mN/m. Zhang and Miller (1992) stated that effective biosurfactants can lower the ST of water to less than 40 mN/m. This ST reduction profile is in agreement with that formerly detected and ascribed to RL production in *P. aeruginosa* (Abdel-Mawgoud et al. 2009; Twigg et al. 2018). The concentration of a surfactant above which micelles or aggregates of surfactant molecule aggregates begin to develop and ST does not drop further is called the CMC. It is an essential character for evaluating the interfacial activity of surface active agents (Zhou et al. 2019). The CMC is obtained from the intersection of the straight line passing through the plateau with the regression straight line of the linearly dependent section (Fig. 4). As shown in the results, the CMC of the tested RLs was 200 mg/L.CMC values for biosurfactants vary from 5 to 386 mg/L (Abbasi et al. 2012). For example, Abalos et al. (2001) showed that different combinations of mono and di-RLs produced had CMC values of 106, 150 and 234 mg/L. Rikalovic et al. (2013) stated that variations in RLs molecular composition such as proportion and structure of congeners, the existence of unsaturated bonds, the branching and length of aliphatic chains of RLs can explain the variable CMC values of RLs produced. A smaller CMC results from a higher mono-/di-RL ratio suggesting that mono-RLs begin micelle formation at concentrations lower than di-RL, but di-RLs have greater bioavailability than mono-RLs (Rikalovic et al. 2013). Also, RLs with less hydrophobic (e.g. short chain) fatty acids form micelles at higher concentrations (Nitschke et al. 2005b; Rikalovic et al. 2013). Pereira et al. (2012) also showed that di-RLs or RLs with short chain fatty acids begin to form micelles at higher concentrations. The RL profile of our mixture, containing high di-RL proportions, explains the chemical basis of the obtained relatively high CMC value. Similar results were reported by Liyanage et al.(2019) who showed that a high di-RLs proportion in their mixtures lead to higher CMC values.

Recently, Kłosowska-Chomiczewska et al. (2017) collected the CMC data of 97 RLs, mainly mixtures, reported in previous articles and evaluated the effect of different factors on the reported CMCs. The feed material, or carbon substrate hydrophobicity was found to be the most influential variable to affect the CMC, where more hydrophilic substrates give di-RLs, with higher CMCs. This may additionally explain the relatively high CMC value obtained in our study, since our substrate was glycerol. Another reason may be due to the purity degree of the RLs, sine the lower the purity of the RLs the higher the CMC values (Kłosowska-Chomiczewska et al. 2017).

These results suggest that the RLs produced by P6 are better alternatives than synthetic surfactants, particularly due to lower effects on the environment. This is because the CMC value of our RL mixture is significantly lower than common chemical surfactants like sodium dodecyl sulphate, SDS (CMC 2200 mg/L) (Khademolhosseini et al. 2019). Hence, less RL is required to reach a maximum reduction in ST, which is why RLs are more effective compared to chemical surfactants.

The emulsification rate is also a unique property of any surfactant. It was therefore evaluated for the produced RLs in the CFS using hexadecane. Results showed that high E24 (63.3% ) was obtained which indicates good emulsifying activity when compared to other studies (Abdel-Mawgoud et al. 2009; Zhao et al. 2018). This good emulsifying activity makes them suitable for use in MEOR. Furthermore, RLs can increase the bioavailability of the hydrocarbons in contaminated sites through emulsification and help in bioremediation (Zhao et al. 2018). They can also be used as oil-mobilizing agents to displace the oil trapped in reservoirs (Gudina et al. 2015).

#### Stability of RLs

RLs are promising bioremediation compounds; so, their stability at different temperatures, saline concentrations, and pH values was studied. To study the effect of different environmental conditions, the aqueous RL solution was diluted to 0.3 g/L. This was the minimum concentration that can cause maximum lowering in water ST (which was determined from Fig. 4), and was therefore selected. A set of RL solutions was then subjected to different environmental conditions and tested for RL activity by OST. Morikawa et al. (2000) and Cheng et al. (2017) confirmed that OST can be reliably used for measuring surface activity of biosurfactants and that its sensitivity is high enough to detect minute amounts of biosurfactants. Several researchers (Abbasi et al. 2012; Khademolhosseini et al. 2019) used OST to evaluate the effect of different conditions on RL activity and showed that any decrease in diameter reflected a change in RL stability. The benefits of this technique are that it is fast and simple, is done using minute volumes of the sample and does not need specialized instruments.

Results showed that temperature had a minor impact on the RL performance over a wide range from 20 to 100 °C. RLs are known to go through stages of precipitation at low temperatures (4 °C) and therefore these temperatures negatively impact surface activity of RLs. After autoclaving, there was nearly no decrease in surface activity. We also investigated the effect of high temperatures on RL emulsifying activity in their crude form (CFS) without the previous expensive steps of extraction. Results showed that autoclaving caused a slight reduction in its emulsification index. The very good thermal stability and great emulsifying activity of the tested RLs makes them appropriate for MEOR and bioremediation of oil-polluted sites where high temperatures exist (Zhou et al. 2019). Moreover, using RLs in their crude form is a plus since it will save costs usually spent on extraction and purification.

The effect of ionic strength on surface activity was also investigated and results showed that surface activity was significantly reduced at NaCl concentrations above 9% w/v. On the other hand, chemical surfactants are inactivated by salt concentration of 2–3% (Abdel-Mawgoud et al. 2009). Therefore, the extracted RLs have proven to be a better choice to be used in the bioremediation of high salinity sites such as contaminated marines.

In case of pH, the best surface activity was obtained at pH 6 to 8. In highly acidic (pH 2) or highly alkaline conditions (pH ≥ 8), a significant reduction in surface activity was noticed. This occurs because RLs are precipitated in strongly acidic conditions, its structure is distorted and its capability to lower ST is reduced. The negatively charged groups at the polar ends of RL biosurfactant molecules become protonated under these acidic conditions (Gogoi et al. 2016). Similar results were reported by several researchers who showed that RLs activity was stable over wide ranges of temperature, salinity and pH (Abdel-Mawgoud et al. 2009; Zhao et al. 2018; Zhou et al. 2019).

Therefore, these results showed that the extracted RLs showed excellent performance over a broad range of temperatures, ionic strengths and pH values, indicating that they can be beneficial in harsh environments. In contrast, SDS was reported to be active within a rather limited range of pH (6–8), salinities (0–8 g% NaCl), and temperatures (25–80 °C) (Cheng et al. 2017). Hence, we concluded that the our extracted RLs were a superior surfactant for use in environmental remediation. Moreover, crude RL products as CFS gave high emulsifying activities after autoclaving, which makes it a good option for immediate application in the oil industry where less purity requirements are needed.

Therefore, in the present study, structural characterization of RLs produced by *P. aeruginosa* P6 using TLC, ^1^H NMR and LC-MS revealed that this isolate, in its wild type form, and using the cheap substrate glycerol, synthesizes a substantially high ratio of di-RLs (di-RLs: mono-RLs: = 80.16: 19.84) which can have promising biotechnological applications. RLs extracted from *P. aeruginosa* P6 resulted in an efficient decrease in water ST, reaching 36 mN/m, characterizing them as powerful surface agents. They were also capable of stabilizing the emulsion formed with hexadecane, reaching an E24 value of 63%. Additionally, RLs remained stable over a broad range of pHs, temperatures and salinities, indicating that they can be used for bioremediation in extreme environments. The excellent qualities of our product, together with the low costs associated with its production and extraction, make it a very cost-effective candidate for various applications in many industries and merit further studies to scale up the production of this remarkable product.

## Data Availability

All data generated or analyzed during this study are included in this published article.

## Abbreviations

RLs: Rhamnolipids
CMC: Critical micelle concentration
ST: Surface tension
E24: Emulsification index
